# The Stepping Threshold Test for assessing reactive balance discriminates between older adult fallers and non-fallers

**DOI:** 10.3389/fspor.2024.1462177

**Published:** 2024-10-11

**Authors:** Natalie Hezel, Theresa Buchner, Clemens Becker, Jürgen M. Bauer, Lizeth H. Sloot, Simon Steib, Christian Werner

**Affiliations:** ^1^ Geriatric Centre, Heidelberg University Hospital, Agaplesion Bethanien Hospital Heidelberg, Heidelberg, Germany; ^2^ Unit of Digital Geriatric Medicine, Heidelberg University Hospital, Heidelberg, Germany; ^3^ Optimization, Robotics, and Biomechanics, Institute of Computer Engineering, Heidelberg University, Heidelberg, Germany; ^4^ Translational and Clinical Research Institute (TCRI), Newcastle University, Newcastle, United Kingdom; ^5^ Department of Human Movement, Training and Active Aging, Institute of Sports and Sports Science, Heidelberg University, Heidelberg, Germany

**Keywords:** reactive balance, postural control, perturbation, falls, risk assessment, older adults, validation study

## Abstract

**Introduction:**

The ability to respond effectively to external perturbations is crucial for avoiding falls. The Stepping Threshold Test (STT) has been developed to assess this reactive balance, but its ability to discriminate between fallers and non-fallers is still unsubstantiated. This study aimed to evaluate the discriminant validity of the STT in distinguishing fallers and non-fallers and its convergent validity.

**Methods:**

Thirty-six older adults (age = 80 ± 5 years), with 13 (36%) of them reporting a fall history in the past year, completed the STT on a perturbation treadmill. They received surface perturbations of progressively increasing magnitude while standing. Single- and multiple-step thresholds were assessed using an all-step count evaluation (STT-ACE), and a direction-sensitive evaluation strategy (STT-DSE). Receiver operating characteristics and area under the curves (AUC) were analyzed to evaluate the discriminative accuracy. Convergent validity was explored by 13 hypothesized associations with other mobility, psychological, and cognitive assessments.

**Results:**

Fallers and non-fallers significantly differed in the STT-DSE (*p* = 0.033), but not in the STT-ACE or other commonly used mobility assessments. Acceptable discriminative accuracy was obtained for the STT-DSE (AUC = 0.72), but not for the STT-ACE and other mobility assessments (AUC = 0.53–0.68). Twelve (92%) associations were consistent with our hypotheses for the STT-DSE, and ten (77%) for the STT-ACE.

**Conclusion:**

Our findings provide preliminary evidence that the STT, when using the STT-DSE, may discriminate between older adult fallers and non-fallers. The STT appears to be a valid tool for assessing reactive balance, with its STT-DSE being recommended due to its better discriminant and convergent validity compared to the STT-ACE.

## 1 Introduction

Falls among older people can be serious incidents that too often lead to personal and social consequences such as avoidance of physical activity, fear of falling, or financial burden for health care. Effective fall prevention involves early identification of individuals at risk of falling. According to the World Falls Guidelines, the assessment of gait and balance is strongly recommended for fall risk assessment (1). Commonly used assessment tools for evaluating gait and balance in relation to fall risk (1, 2) include gait speed tests (3), Timed Up and Go (TUG) (4), Berg Balance Scale (BBS) (5), 5-chair stand test (3), and Short Physical Performance Battery (SPPB) (3). However, previous studies have indicated that most of these tools are inconsistent in their ability to predict or differentiate between older adult fallers and non-fallers (1, 2, 6, 7).

Reactive balance, defined as the ability to respond effectively to external perturbations (e.g., slips, trips) in order to avoid a loss of balance (8), plays a crucial role in the multifactorial aetiology of falls. Notably, slipping and tripping are the most common circumstances in which falls occur among older adults (9–13). As the purpose of fall risk assessment is to address the mechanism of falls and contributing risk factors (1), reactive balance should be an integral part of evaluating an individual's risk of falling (14, 15). However, most frequently used mobility assessments that aim to evaluate fall risk such as gait speed tests, TUG, BBS or SPPB do not measure reactive balance abilities. In contrast, the Balance Evaluation Systems Test (BESTest) (16) or the Performance-Oriented Mobility Assessment (POMA) (17), two other established gait and balance assessment tools, contain single items on reactive balance. However, they have a limited ability to specifically assess reactive balance since the few reactive balance items in both tests have a low level of unpredictability concerning unexpected loss of balance, which is crucial for testing reactive balance (18). To date, specific tests on reactive balance are rare and usually consist of waist-pulls, tether-releases, or platform motions (15). These methods may, however, also be constrained by one-directional perturbations, low unpredictability, difficulty in adjusting the perturbation magnitude, and/or a narrow focus on only reactive stepping rather than overall reactive balance—all of which have been mentioned as important aspects for assessing reactive balance (15, 18–20). Recent developments in treadmill technologies enable the application of perturbations in various directions, with a precise adjustment of their magnitude and high level of unpredictability (21).

The Stepping Threshold Test (STT) (22) has recently been developed for specific reactive balance assessment on a perturbation treadmill that evaluates compensatory stepping responses to unexpected surface perturbations of increasing magnitude in anterior-posterior (AP) and medio-lateral (ML) directions. The convergent validity of the STT has been documented in fall-prone older adults by testing hypotheses on associations with measures of global and static balance (Brief-BESTest, 8-level balance scale), functional mobility (TUG), and fear of falling [Short Falls Efficacy Scale-International (Short FES-I)] (22). In addition, the STT evaluation strategy in classifying reactive balance responses has been proven to be inter-observer reliable in healthy adults and stroke patients, and convergently valid (BBS, 6-min walk test, FES-I) in stroke patients (23). However, there is still a lack of evidence on the discriminant validity of the STT in distinguishing between fallers and non-fallers, and its convergent validity has not yet been demonstrated through expected associations with other measures on reactive balance, gait capacity, muscle strength, and executive and cognitive functioning.

Thus, the aim of this study was to evaluate (1) the discriminant validity of the STT to distinguish between older adult fallers and non-fallers, and (2) the convergent validity of the STT by testing 13 hypotheses on associations with various fall risk factors. We hypothesized that older adults with a fall history would show significant differences in the STT compared to those without. Furthermore, we expected the STT, as a measure of reactive balance, to show moderate positive associations with global (22, 24, 25), static (25, 26), and dynamic (reactive) balance (27, 28), gait capacity (23, 24, 26), functional mobility (22, 25), global cognition (29), and executive functioning (30), as well as low to moderate positive associations with muscle strength (26, 30), and a moderate negative association with fear of falling (24, 26, 31).

## 2 Methods

### 2.1 Design

This is a secondary analysis of baseline data from the FEATURE study, a randomized, controlled pilot intervention trial that evaluates the effects of perturbation-based treadmill training on reactive balance in fall-prone older adults. Details about the design, intervention, and primary outcomes of this study have been described previously (32). The FEATURE study was approved by the Ethics Committee of the Medical Faculty Heidelberg (S-602/2022), conducted in accordance with the Helsinki Declaration, and prospectively registered at the German Clinical Trials Register (DRKS00030805). Written informed consent was obtained from all participants prior to study inclusion.

### 2.2 Participants

Participants were recruited between January and July 2023 from an ambulatory geriatric rehabilitation sports club (REGE e.V.) for older adults, that is associated with a German geriatric hospital. Inclusion criteria were age ≥65 years, increased risk of falling [TUG >12 s and/or usual gait speed <1.0 m/s, and/or fall(s) in past 12 months], and ability to walk ≥2 min without walking aid. Exclusion criteria were cognitive impairment [Mini-Mental State Examination (MMSE) <24 pt.] (33), or severe neurological, cardiovascular, metabolic, and psychiatric disorders.

### 2.3 Measurements

All measurements were consistently administered by a master’s student in sports science (C.L.), who had received extensive training in interview and test administration to ensure the highest possible standardization and data quality.

#### 2.3.1 Descriptive measures

Age, gender, chronic disease, education, and fall history (≥1 fall in the past 12 months) were assessed by self-reporting. A fall was defined as any “unexpected event in which the participant comes to rest on the ground, floor, or lower level” (34), without considering whether the fall was avoidable or not. Nutritional status was assessed through the body mass index. Cognitive measures included the MMSE (33) for global cognition and the Trail Making Test (TMT) (35) for executive functioning. Subjective health status was assessed using the EQ-5D visual analogue scale (36). Psychological measures comprised the Short FES-I (37) and 5-item Geriatric Depression Scale (GDS-5) (38, 39). Physical frailty was determined according to the criteria of the Fried frailty phenotype (unintentional weight loss, exhaustion, low physical activity, slowness, weakness) (40).

#### 2.3.2 Mobility assessments

Other balance measures included the Brief-BESTest (41) for global balance, the SPPB balance test (3) for static balance, the Four Square Step Test (FSST) (42) for dynamic balance, and the Dynamic Stepping Threshold Test (DSTT) for dynamic reactive balance, which is a modified version of the STT while walking (32). Muscle strength was assessed by a handgrip strength test (JAMAR dynamometer) (43) and the 5-chair stand test (3). Functional mobility was measured with the SPPB (3) and TUG (4), and gait capacity with the 4-m gait speed test (3) and 2-min walk test (2MWT) (44).

#### 2.3.3 Stepping Threshold Test

The STT assesses reactive balance while standing and is executed on a perturbation treadmill that allows for surface translation perturbations in AP and ML directions with increasing magnitude (22). Participants are secured by a harness system and instructed to stand with both feet together, reacting to a maximum of 24 (+1) unannounced perturbations by as few compensatory steps as possible. The test consists of six levels with four perturbations each (forward, backward, left, right) in random order and with gradually increasing magnitudes over the six levels (Supplementary Material). Time intervals between perturbations range from 10 to 19.5 s and are also randomized. Level 4 contains an additional perturbation not considered for the evaluation to maintain the unpredictability of the perturbation direction. The STT total score (8–56 pt.) is the sum of eight single-step and multiple-step thresholds (2 thresholds×4 directions), each defined as the level (1–6) at which a participant requires one step or multiple steps (≥2) to regain balance. The stepping behavior can be evaluated using an all-step count evaluation (STT-ACE) and a direction-sensitive evaluation strategy. For the STT-ACE, each step—defined as an observable change in the bipedal base of support (BoS)—is counted until the participant reaches a static steady-state balance after the perturbation. For the STT-DSE, only steps that result in a sensible extension of the BoS in the opposite direction of the surface translation perturbation are considered (22). Thresholds have to be confirmed in two consecutive levels to be scored while the first of these levels is set as the threshold (45). The STT prematurely is terminated if participants fall or express excessive fear demanding the test to be stopped. If so, missing thresholds are set at one level above the last executed level (22). The STT was performed on the BalanceTutor™ (MediTouch, Netanya, Israel) and video recorded by two cameras (HERO9 Black, GoPro, San Mateo, CA, USA) positioned at about 35° fronto-lateral to the participant and recording at a frame rate of 60 Hz. The video-based assessment of step thresholds in response to surface perturbations during standing has been shown to be inter-observer reliable (Kappa coefficient = 0.89–0.99) (23). Step thresholds for STT scoring were determined from the video recordings by the same rater (N.H.) for consistency, who was not involved in the descriptive measures and mobility assessments.

### 2.4 Assessment of discriminant and convergent validity

Discriminant validity—defined as the ability of an instrument to differentiate between groups that are known to be different (46)—was evaluated by the discriminative accuracy of the STT-ACE and STT-DSE to distinguish between participants with fall history (fallers) and those without (non-fallers). The discriminative accuracy of other commonly used mobility assessments for fall risk was also examined to provide a preliminary basis for comparison.

Convergent validity—defined as the extent to whether the instrument under study correlates with other instruments to the degree one would expect (47)—was examined through testing 13 hypotheses on expected associations of the STT-ACE and STT-DSE, respectively, with other mobility, psychological and cognitive fall risk factors. The correlation hypotheses are based on existing literature (see introduction). As recommended in guidelines for evaluating psychometric properties (47, 48), convergent validity was considered to be established if ≥75% of the observed associations were consistent with our hypotheses.

### 2.5 Statistical analysis

Differences between fallers and non-fallers were analyzed using *t*-tests for independent samples, Mann-Whitney *U*-tests, and χ^2^-tests or Fisher's exact tests. Receiver operating characteristic (ROC) and area under the curve (AUC) analyses were performed to determine the accuracy and optimal cutoff points of each mobility assessment in discriminating fallers from non-fallers. AUC values were interpreted as follows: no (AUC ≤0.5), poor (0.5< AUC <0.7), acceptable (0.7≤ AUC <0.8), excellent (0.8≤ AUC <0.9), or outstanding discriminative (AUC ≥0.9) (49). Youden's index [(sensitivity + specificity)–1] was calculated to identify the optimal cutoff point from ROC analyses (50). Spearman rank correlation coefficients (*r*) were calculated for testing the correlation hypotheses of the STT-ACE and STT-DSE with other mobility, psychological, and cognitive assessments. Coefficients were interpreted as low (*r* < 0.3), moderate (*r* = 0.3–0.5), or high (*r* > 0.5) (51). Scatter plots with regression lines and 95%-confidence interval (CI) bands were also constructed to visualize correlations. Statistical significance was set at *p* < 0.05. All statistical analyses were performed using IBM SPSS version 29.0 (IBM Corporation, Armonk, NY, USA).

## 3 Results

### 3.1 Participant characteristics

Thirty-six community-dwelling older adults (age = 80.3 ± 5.4 years, females: *n* = 26, 72.2%) were included. One third (*n* = 13, 36%) reported experiencing at least one fall in the past 12 months (Table 1), with only 4 participants (11%) classified as recurrent fallers (≥2 falls). More than half (*n* = 19, 53%) were categorized as pre-frail or frail. Gait capacity was mildly impaired, with a mean gait speed of 0.84 ± 0.16 m/s (Table 2), and two-thirds reported at least moderate fear of falling (*n* = 24, 66.7%). No significant differences in sociodemographic, nutritional, medical, cognitive, psychological, and frailty characteristics were observed between fallers and non-fallers (*p* = 0.162–0.568; Table 1). Two participants, one with and one without fall history, declined to conduct the STT due to anxiety.

**Table 1:** Participant characteristics.

Variable	Total (*n* = 36)	Fallers (*n* = 13)	Non-Fallers (*n* = 23)	*p*
Age, years	80.3 ± 5.4	81.2 ± 7.4	79.8 ± 3.9	0.568
Females, *n*	26 (72.2)	8 (61.5)	18 (78.2)	0.440
Chronic disease, *n*	30 (83.3)	12 (92.3)	18 (78.3)	0.385
Education, years	13.1 ± 3.4	14.0 ± 3.4	12.7 ± 3.4	0.270
BMI, kg/m^2^	25.9 ± 3.7	24.9 ± 3.4	26.5 ± 3.8	0.220
BMI categories, *n*				0.529
Underweight (<23 kg/m^2^)	6 (16.7)	3 (23.1)	3 (13.0)	
Normal (23–30 kg/m^2^)	24 (66.7)	9 (69.2)	15 (65.2)	
Overweight (>30 kg/m^2^)	6 (16.7)	1 (7.7)	5 (21.7)	
MMSE, pt.	28.2 ± 1.5	27.9 ± 1.6	28.4 ± 1.3	0.250
TMT B-A, s	79.4 [43.3–117.2]	91.0 [37.5–131.7]	59.0 [42.9–108.7]	0.564
EQ-5D VAS, pt.	72.7 ± 17.2	76.9 ± 14.9	70.4 ± 18.2	0.276
GDS-5, pt.	1 [0–1]	0 [0–1]	1 [0–1]	0.426
Short FES-I, pt.	9.5 [8–11]	10 [9–12]	9 [8–10]	0.162
Fear of falling, *n*				0.530
Low	12 (33.3)	3 (23.1)	9 (39.1)	
Moderate	22 (61.1)	9 (69.2)	13 (56.5)	
High	2 (5.6)	1 (7.7)	1 (4.3)	
Physical frailty, *n*				0.217
Robust	17 (47.2)	4 (30.8)	13 (56.5)	
Pre-frail	16 (44.4)	7 (53.8)	9 (39.1)	
Frail	3 (8.3)	2 (15.4)	1 (4.3)	

Descriptive data given as *n* (%), median [interquartile range], or mean ± standard deviation. BMI, body mass index; MMSE, Mini Mental State Examination; TMT, Trail Making Test; EQ-5D, quality of life questionnaire; VAS, visual analogue scale; GDS-5, 5-item Geriatric Depression Scale; Short FES-I, Short Falls Efficacy Scale-International. Fear of falling according to Short FES-I: low = 7–8 pt., moderate = 9–13 pt., high, ≥14 pt., and physical frailty according to Fried's frailty phenotype: robust = 0–1 pt., pre-frail = 2 pt., frail = 3–5pt. *P*-values given for *t*-tests for independent samples (age, education, BMI, MMSE, EQ-5D VAS), Mann-Whitney *U-*tests (TMT B-A, GDS-5, Short FES-I), or χ^2^-tests or Fisher's exact tests (females, BMI categories, fear of falling, physical frailty).

**Table 2:** Differences in mobility assessments between fallers and non-fallers.

Variable	Total (*n* = 36)	Fallers (*n* = 13)	Non-Fallers (*n* = 23)	*p*	AUC (95%-CI)	Cutoff point	Sensitivity	Specificity
Reactive balance^a^								
STT-ACE, pt.	17.0 [13.5–20.0]	14.5 [11.5–19.3]	17.0 [14.8–22.3]	0.086	0.68 (0.49; 0.88)	15.5	0.67	0.73
STT-DSE, pt.	21.0 [17.0–27.0]	17.0 [14.3–25.5]	21.0 [17.8–29.0]	0.033	0.72 (0.54; 0.91)	15.5	0.96	0.42
Global balance								
Brief-BESTest, pt.	12.0 [9.0–17.0]	9.0 [6.0–16.0]	12.0 [10.0–17.5]	0.177	0.64 (0.44; 0.84)	9.5	0.54	0.81
Static balance								
SPPB balance test, pt.	3 [3–4]	3 [3–4]	4 [3–4]	0.129	0.64 (0.45; 0.83)	3.5	0.62	0.65
Dynamic balance								
FSST, s	10.8 [9.0–12.9]	12.4 [9.1–20.2]	10.5 [9.2–13.1]	0.420	0.58 (0.37; 0.79)	11.6	0.62	0.65
Muscle strength								
Handgrip strength, kg	22.4 [19.5–31.5]	21.7 [19.4–36.5]	24.7 [20.3–29.6]	0.754	0.53 (0.32; 0.74)	21.9	0.54	0.70
5-chair stand test, s	11.3 [9.6–13.4]	11.8 [9.6–13.6]	11.1 [9.1–12.7]	0.482	0.57 (0.37; 0.78)	12.3	0.50	0.73
Functional mobility								
SPPB, pt.	11.0 [9.5–12.0]	10.0 [7.5–11.5]	11.0 [10.0–12.0]	0.205	0.63 (0.43; 0.83)	8.5	0.39	0.91
TUG, s	10.6 [8.5–13.7]	11.6 [8.3–15.9]	10.3 [8.9–13.6]	0.542	0.56 (0.35; 0.77)	14.3	0.39	0.87
Gait capacity								
4-m gait speed test, m/s	0.84 ± 0.16	0.79 ± 0.22	0.87 ± 0.12	0.113	0.60 (0.39; 0.82)	0.72	0.39	0.96
2MWT, m	122.6 ± 30.9	108.4 ± 36.7	126.6 ± 28.8	0.110	0.65 (0.45; 0.85)	114.4	0.62	0.74

Descriptive data given as median [interquartile range] or mean ± standard deviation. AUC, area under the curve; CI, confidence interval; STT, Stepping Threshold Test; ACE, all-step count evaluation; DSE, direction-sensitive evaluation; Brief-BESTest, Brief Balance Evaluation Systems Test; SPPB, Short Physical Performance Battery; FSST, Four Square Step Test; TUG, Timed Up and Go; 2MWT, 2-min walk test.

^a^ Based on *n* = 34 (fallers: *n* = 12, non-fallers: *n* = 22). *P*-values given for Mann-Whitney *U*-tests (STT-ACE, STT-DSE, Brief-BESTest, SPPB static balance, FSST, handgrip strength, 5-chair stand test, SPPB, TUG) or *t*-tests for independent samples (4-m gait speed test, 2MWT).

### 3.2 Discriminant validity

**Figure 1:**
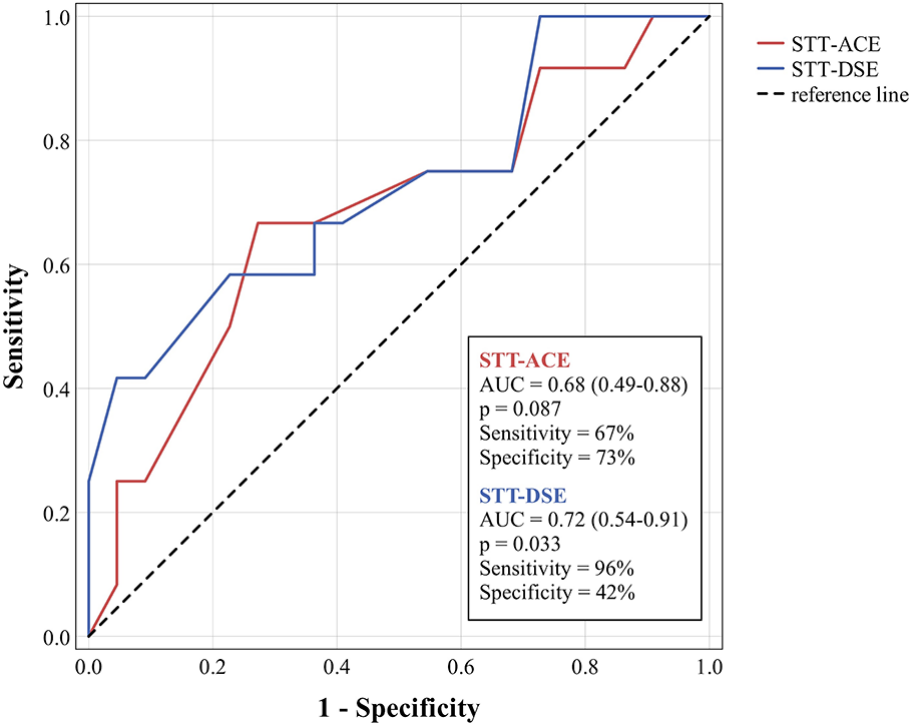
Receiver operating characteristic curves for discriminating fallers and non-fallers using the STT-ACE and STT-DSE.

The STT-DSE score differed significantly between fallers and non-fallers (*p* = 0.033) with acceptable discriminative accuracy [AUC = 0.72, 95%-confidence interval (CI) 0.54–0.91]. The optimal cutoff point for the STT-DSE was 15.5 points, with 96% sensitivity and 42% specificity (Figure 1). No significant differences between fallers and non-fallers (*p* = 0.086–0.754) and poor discriminative accuracy (AUC = 0.53–0.68, 95%–CI 0.32–0.88) were found for the STT-ACE (AUC = 0.68, 95%–CI 0.49–0.88) and all other mobility assessments (AUC = 0.53–0.65, 95%–CI 0.32–0.85) (Table 2).

### 3.3 Convergent validity

Twelve out of 13 observed associations (92%) with global, static, dynamic and reactive balance, gait capacity, muscle strength, functional mobility, global cognition and executive functioning were consistent with our hypotheses for the STT-DSE (Table 3). The only association for the STT-DSE that was not aligned with our hypotheses was fear of falling. Ten out of 13 observed associations (77%) were consistent for the STT-ACE, with fear of falling, global cognitive, and executive functioning not aligning as expected. High correlations were found for both STT evaluation strategies with global, static, and dynamic reactive balance (BriefBESTest, SPPB balance test, DSTT: *r*≥0.53), and moderate to high correlations with dynamic balance (FSST: *r*=–0.37;–0.52), gait capacity (4-m gait speed test, 2MWT: *r* = 0.38;0.62), and functional mobility (TUG, SPPB: *r*=|0.34;0.55|). Low correlations were observed for fear of falling (Short FES-I: *r*≤|0.28|). Correlations with cognitive measures were moderate for the STT-ACE (MMSE, TMT: *r*≥|0.39|), but low for the STT-DSE (MMSE, TMT: *r*≤|0.29|). Except for muscle strength (5-chair stand test), the STT-DSE (*r*=|0.23–0.69|) demonstrated higher correlations than the STT-ACE (*r*=|0.18–0.58|) with the other assessments. Scatter plots for all correlations are presented in the Supplementary Material.

**Table 3:** Associations of the two evaluation strategies of the Stepping Threshold Test with other assessments of fall risk factors.

No.	Hypothesis	Instrument	STT-ACE			STT-DSE		
			*r* (95%-CI)	*p*	Consistent with hypothesis	*r* (95%-CI)	*p*	Consistent with hypothesis
1	Positive association of at least moderate strength (*r*≥0.30) between STT and global balance (22, 24, 25).	Brief-BESTest	0.56 (0.26; 0.77)	<0.001	Yes	0.69 (0.44; 0.84)	<0.001	Yes
2	Positive association of at least moderate strength (*r*≥0.30) between STT and static balance (25, 26).	SPPB balance test	0.53 (0.22; 0.74)	0.001	Yes	0.64 (0.37; 0.81)	<0.001	Yes
3	Positive association of at least moderate strength (*r*≥|0.30|) between STT and dynamic balance (27).	FSST	−0.37 (–0.64; –0.03)	0.030	Yes	–0.52 (–0.73; –0.20)	0.002	Yes
4	Positive association of at least moderate strength (*r*≥0.30) between STT and dynamic reactive balance (28).	DSTT	0.58 (0.29; 0.77)	<0.001	Yes	0.68 (0.43; 0.83)	<0.001	Yes
5	Positive association of at least moderate strength (*r*≥0.30) between STT and gait capacity (23, 24, 26).	4-m gait speed test	0.38 (0.04; 0.64)	0.028	Yes	0.44 (0.11; 0.68)	0.009	Yes
6		2MWT	0.50 (0.18; 0.72)	0.003	Yes	0.62 (0.34; 0.79)	<0.001	Yes
7	Positive association of low to moderate (|0.10|<*r*≥|0.30|) strength between STT and muscle strength (26, 30).	Handgrip strength	0.18 (–0.17; 0.48)	0.306	Yes	0.23 (–0.12; 0.54)	0.185	Yes
8		5-chair stand test	–0.22 (–0.53; 0.15)	0.235	Yes	–0.16 (–0.49; 0.21)	0.378	Yes
9	Positive association of at least moderate strength (*r*≥|0.30|) between STT and functional mobility (22, 25).	SPPB	0.34 (0.00; 0.62)	0.047	Yes	0.45 (0.12; 0.69)	0.008	Yes
10		TUG	–0.45 (–0.69; –0.12)	0.008	Yes	–0.55 (–0.76; –0.26)	<0.001	Yes
11	Negative association of at least moderate strength (*r*≤–0.30) between STT and fear of falling (24, 26, 31).	Short FES-I	–0.18 (–0.50; 0.18)	0.300	No	–0.28 (–0.57; 0.08)	0.113	No
12	Positive association of at least moderate strength (*r*≥0.30) between STT and global cognition (29).	MMSE	0.25 (–0.11; 0.55)	0.158	No	0.46 (0.13; 0.69)	0.007	Yes
13	Positive association of at least moderate strength (*r*≥|0.30|) between STT and executive functioning (30).	TMT B-A	–0.29 (–0.58; 0.06)	0.091	No	–0.38 (–0.64; –0.04)	0.027	Yes

Correlation coefficients (*r*) are presented as Spearman rank correlations with 95%-confidence intervals (CI). STT, Stepping Threshold Test; ACE, all-step count evaluation; DSE, direction-sensitive evaluation; FSST, Four Square Step Test; DSTT, Dynamic Stepping Threshold Test; SPPB, Short Physical Performance Battery; Brief-BESTest, Brief Balance Evaluation Systems Test; 2MWT, 2-min walk test; TUG, Timed Up and Go; Short FES-I, Short Falls Efficacy Scale-International; TMT B-A, difference between part B and A of the Trail Making Test; MMSE, Mini Mental State Examination.

## 4 Discussion

This study evaluated the discriminant and convergent validity of the STT in community-dwelling, fall-prone older adults. To our knowledge, our findings are the first to show acceptable discriminative accuracy of the STT in distinguishing older adult fallers from non-fallers through its DSE strategy, which considers only those compensatory steps in the opposite direction of the perturbation. The ACE strategy of the STT and other commonly used mobility assessments for fall risk showed only poor discriminative accuracy in our sample. Convergent validity of the STT was suggested by 92% of the observed associations for the STT-DSE and 77% for the STT-ACE with various fall risk factors being consistent with our hypotheses, indicating that the STT-DSE might be more valid than the STT-ACE. The results extend previous findings (22) on the convergent validity of the STT in fall-prone older adults by investigating associations with measures of dynamic reactive balance, gait capacity, muscle strength, and cognitive and executive functioning.

### 4.1 Discriminant validity

The initial STT development and validation study by Adams et al. (22) also investigated its discriminant validity but found no significant differences between fallers and non-fallers using either STT evaluation strategy. In contrast, we found a significantly lower STT-DSE score for fallers, demonstrating acceptable discriminative accuracy (AUC = 0.72) for identifying participants’ fall history. The difference in the STT-ACE came close to the level of significance (*p* = 0.086) and to the threshold for acceptable discriminative accuracy (AUC = 0.68). The discrepancy to Adams et al. (22) might have resulted from the higher heterogeneity in STT scores [robust coefficient of variance (RCV) = 53%–66%] within our older sample (age = 80 ± 5 years) as opposed to their younger sample (age = 75 ± 6 years; RCV = 21%–33%). Our findings, however, align with those of a meta-analysis on stepping performance and falls in older adults that showed fallers performing worse in reactive step tests (e.g., waist-pulls, tether-releases, slip perturbations) and taking more recovery steps following a perturbation compared to non-fallers (15). These reactive step tests showed moderate sensitivity (73%, 95%–CI 57%–85%), low specificity (59%, 95%–CI 33%–81%), and an acceptable AUC (0.74, 95%–CI 0.47–0.90). Further, more recent original studies confirmed the lower reactive balance ability of older adult fallers (31, 52). Crenshaw et al. (52) performed AP surface perturbations while standing and found that the posterior single-step threshold was lower in fallers, with a significant, but poor discriminative accuracy between fallers and non-fallers (*p* = 0.049, AUC = 0.62). Batcir et al. (31), who applied surface perturbations in ML direction while standing, also showed that single-step and multiple-step thresholds were lower in fallers.

The STT-DSE seemed to be slightly more accurate compared to the STT-ACE in discriminating fallers from non-fallers in our study. These two evaluation strategies offer different perspectives on stepping behavior. Reactive steps in the opposite direction of the surface perturbation, as considered in the STT-DSE, extend the BoS toward the center of mass (COM) motion to keep the COM within the limits of stability. These (crossover) steps directly contribute to maintaining balance and are effective reactive stepping behaviors for preventing falling, whereas ineffective crossover stepping has been associated with unsuccessful balance recovery in older adults in response to lateral surface perturbations while standing (53). Other steps extending the BoS in different directions, as considered in the STT-ACE, may be rather ineffective and not directly relevant to balance control, or might merely serve to increase standing comfort (22). Thus, the STT-DSE may provide a more accurate mapping of reactive balance ability compared to the STT-ACE. The general assumption that fallers have poorer reactive balance than non-fallers supports our findings that the STT-DSE showed acceptable discriminant validity and might be suitable for evaluating an individual's reactive balance and fall risk. In addition, especially in more frail populations with limited overall physical capacity, the STT-DSE might be more appropriate, as these individuals may take more “ineffective” steps that are not relevant for directly compensating for balance disturbances but rather for improving standing comfort, which are nonetheless considered in the STT-ACE.

Fallers and non-fallers did not show significant differences in other mobility assessments for fall risk, which also consistently demonstrated only poor discriminative accuracy (AUC = 0.53–0.65). This could be attributed to the fact that these assessments do not specifically address common fall mechanisms in real life (e.g., unexpected trips or slips), which may result in lower ecological validity compared to reactive balance measures such as the STT. Notably, previous studies in larger samples of community-dwelling older adults have shown higher discriminative accuracy for some of these mobility assessments (e.g., Brief-BESTest: AUC = 0.76, gait speed test: AUC = 0.69, TUG: AUC = 0.80, FSST: AUC = 0.73) (2, 41, 42). Future studies specifically designed to assess differences in the discriminant validity of the STT compared to such assessments, with larger sample sizes, are needed to further explore our preliminary findings.

Our findings suggest that the STT-DSE can distinguish fallers from non-fallers with high sensitivity (96%) and low specificity (42%). Although having both high sensitivity and high specificity is ideal, a fall risk assessment with high sensitivity but low specificity ensures that those truly at risk of falling are accurately identified. This allows for timely fall prevention measures, even if some individuals with low fall risk are also targeted.

### 4.2 Convergent validity

Convergent validity of the STT and its two evaluation strategies was investigated by testing hypotheses on associations reported in previous studies between reactive balance or stepping tests and other mobility-related, psychological, and cognitive fall risk factors. More than 75% of these associations were consistent with our hypotheses for both evaluation strategies, suggesting the STT is convergently valid (47, 48).

Adams et al. (22) observed low to moderate correlations of the STT with measures of global balance (Brief-BESTest: STT-ACE: *r* = 0.41, STT-DSE: *r* = 0.39), static balance (8-level balance scale: STT-ACE: *r* = 0.17, STT-DSE: *r* = 0.25), functional mobility (TUG: STT-ACE: *r* = –0.38, STT-DSE: *r* = –0.44), and fear of falling (Short FES-I: STT-ACE: *r* = –0.11, STT-DSE: *r* = –0.10) in fall-prone older adults (age = 75 ± 6 years; TUG = 7.8 ± 1.3 s). Other studies including people at a supposedly higher risk of falling, such as stroke patients [mean BBS <45 pt. indicate high fall risk (54)] (23, 24), older women (age = 77 ± 8 years) (26) or older adults (age = 79 ± 5 years) (31), and assisted living residents (age = 82 ± 6 years, TUG = 13.8 ± 5.3 s) (25) reported higher correlations of reactive balance tests with global balance (BBS: *r* = 0.44–0.69), static balance (unipedal stance test: *r* = 0.40–0.57), functional mobility: (TUG: *r* = –0.45, POMA: *r* = 0.44), and fear of falling (FES-I: *r* = –0.30 to −0.58). As our population was older (80 ± 5 years) and physically more limited (TUG = 12.6 ± 6.3 s) compared to that of Adams et al. (22), we expected to find also higher correlations between the STT and these fall risk measures. Indeed, we found moderate to high correlations of the STT, irrespective of the evaluation strategy, with the Brief-BESTest, SPPB balance test, TUG, and SPPB (*r* = |0.34–0.69|), being consistent with our hypothesis that the STT is moderately to highly associated with global and static balance, and functional mobility.

The unexpected low correlation with the Short FES-I may be due to the lack of variance in our sample, with only two participants (6%) reporting high fear of falling and a relatively low RCV (33%) in the Short FES-I. In contrast, Handelzalts et al. (23), who correlated the FES-I with an STT-derived fall threshold (i.e., perturbation magnitude that led to unambiguous support by the harness system) reported high correlations (*r* = –0.58) in stroke patients. However, more than half of these patients had high fear of falling, and the RCV of the FES-I was three times as large (100%). Likewise, Batcir et al. (31) observed higher correlations between single-step (*r* = −0.40) and multiple-step (*r* = −0.30) thresholds to ML surface perturbations in older adults, with a high variance in the FES-I (CV >78%). Our findings are consistent with Adams et al. (22), showing low correlations of the Short FES-I with the STT-ACE (*r* = –0.11) and STT-DSE (*r*=–0.10) in fall-prone older adults with similar low variance in the Short FES-I (RCV = 28%).

Werth et al. (27) found a moderate to high correlation (*r* = 0.36–0.52) between reactive and voluntary stepping responses in a mixed sample of young, middle-aged, and older adults. Accordingly, we hypothesized at least moderate correlations between the STT and the FSST as measure of dynamic balance control during volitional stepping. Correlation coefficients of *r*=–0.37 for the STT-ACE and *r*=–0.52 for the STT-DSE with the FSST were consistent with our hypothesis.

Owings et al. (28) reported a moderate correlation (*r* = 0.36) between static reactive balance, measured by a tether-release test (maximum angle for single-step threshold), and dynamic reactive balance, measured by successful balance recovery during walking (not touching treadmill handles or falling into the harness system) in response to an anterior surface perturbation, in healthy older adults. Based on this finding, we hypothesized that the STT would be at least moderately correlated with the DSTT. Correlations for both the STT-ACE (*r* = 0.58) and STT-DSE (*r* = 0.68) aligned with this hypothesis, suggesting even stronger associations. This might be due to the DSTT being a modified version of the STT for measuring reactive balance while walking, with a very similar test protocol that includes perturbations of progressively increasing magnitude and assesses the ability to adapt and learn from repeated perturbations, which has been linked to a lower risk of falling (55, 56).

Higher correlations between the STT and DSTT, compared to the FSST, might be attributed to the fact that reactive balance tests, whether static or dynamic, share more similar control mechanisms with each other than with volitional stepping tests. While both reactive and volitional stepping require fast swing phase and appropriate foot placement, volitional stepping involves more deliberate initiation, unlike the reflexive nature of reactive stepping.

Associations between single-, multiple-step, and/or fall thresholds to surface perturbations while standing and gait capacity measures have been reported to be moderate in community-dwelling older women (7.5-m gait speed test: mean *r* = 0.30, range 0.24–0.37) (26), and moderate to high (10-m gait speed test: mean *r* = 0.34, range 0.12–0.55; 6-min walk test: *r* = 0.60) in stroke patients (23, 24). Thus, we expected at least moderate correlations between the STT and gait capacity also in our study, and the observed correlations for both the STT-ACE and STT-DSE were consistent with this expectation (*r* = 0.38–0.62).

Lower limb muscle strength has been identified as a predictor of step thresholds to anterior and ML waist pulls while standing in older adults (80 ± 4 years) with low to moderate fall risk (30). In addition, another study in older women reported low to moderate correlations of lower limb muscle strength (*r* = 0.15–0.36) and handgrip strength (*r* = 0.20–0.30) with single-step and multiple-step thresholds to AP surface perturbations while standing (26). As hypothesized from these findings, we observed that the STT has only a low correlation with handgrip strength and 5-chair stand test (*r*=|0.16–0.23|).

Few studies have investigated associations between reactive balance and cognitive functioning in older adults. Kannan and Bhatt (29) indicated that cognitively impaired older adults exhibit a deteriorated reactive standing balance control in response to posterior surface perturbations compared to cognitively intact counterparts. Additionally, Sturnieks et al. (30) identified executive functioning (TMT) as predictor of step thresholds to ML waist pulls while standing in older adults. Based on these findings, we hypothesized that the STT would be at least moderately correlated with measures of global cognition (MMSE) and executive functioning (TMT). Correlations observed for the STT-DSE (MMSE: *r* = 0.46, TMT: *r*=–0.39) align with this hypothesis, while those for the STT-ACE (MMSE: *r* = 0.25, TMT: *r*=–0.29) did not.

This study has some limitations. First, as this was a secondary analysis of the FEATURE study, the sample size was small and not specifically designed to evaluate the discriminant and convergent validity of the STT, and the hypotheses for testing its convergent validity were not formulated *a priori*. A sensitivity power analysis was conducted to determine the minimum effect sizes that could be reliably detected by each statistical test used in this study, based on the available sample sizes (Supplementary Material). This analysis revealed that most effect sizes did not meet these thresholds, indicating that some results may be underpowered and need to be interpreted with caution. Future studies with larger sample sizes are required to provide more precise effect size estimates and reduce the risk of type II errors (false negatives), particularly for smaller effect sizes that were consistent with hypotheses in direction and magnitude but did not reach statistical significance. Second, participants were recruited from a geriatric rehabilitation sports club, which may limit the generalizability to the broader older population who may not be regularly engaging in physical exercise. Third, fall history was gathered through self-reporting, which may be subject to recall bias, potentially leading to inaccuracies in the fall history (57). Fourth, as this study was based on a retrospective fall history, the predictive value of the STT cannot be assumed. Future prospective studies are necessary to evaluate the STT's ability to predict future falls.

## 5 Conclusion

The present study provides preliminary evidence for the discriminant and convergent validity of the STT for measuring reactive balance in fall-prone older adults. When using the STT-DSE strategy, the STT showed an acceptable ability to discriminate between older adult fallers and non-fallers. Convergent validity of the STT was suggested by various associations with other mobility-related, psychological, and cognitive fall risk factors that were consistent with our hypotheses, with the STT-DSE appearing to be more convergently valid than the STT-ACE. The STT seems to be an appropriate tool for assessing reactive balance and fall risk in older adults, with its STT-DSE being recommended due to its better discriminant and convergent validity compared to the STT-ACE. Given the exploratory nature of this secondary analysis study and its limited statistical power, the findings should be interpreted with caution. Future studies specifically designed to evaluate the psychometric properties of the STT, with larger sample sizes, are necessary to confirm these findings.

## Data Availability

The raw data supporting the conclusions of this article will be made available by the authors, without undue reservation.
