# Supplementary material for: The Stepping Threshold Test for assessing reactive balance discriminates between older adult fallers and non-fallers
Source: Front Sports Act Living. 2024 Oct 11;6:1462177. doi: 10.3389/fspor.2024.1462177 (PMC11502312; doi:10.3389/fspor.2024.1462177)
Supplement: Supplementary file 1 [file Table1.docx]

Supplementary Material

**Supplement 1: Test protocol for the Stepping Threshold Test**

| **STT level** | **Direction of**  **surface translation** | **Acceleration (cm/s^2^)** | **Displacement (cm)** | **Time intervals**  **between perturbations (s)** |
| --- | --- | --- | --- | --- |
| *Level 1* | AP: Forward | 64 | 7.4 | 16.5 |
|  | ML: Left | 43 | 3.3 | 12.5 |
|  | ML: Right | 43 | 3.3 | 10.0 |
|  | AP: Backward | 64 | 7.4 | 10.0 |
| *Level 2* | ML: Right | 80 | 6.3 | 11.5 |
|  | AP: Forward | 128 | 12.9 | 10.5 |
|  | ML: Left | 80 | 6.3 | 11.5 |
|  | AP: Backward | 128 | 12.9 | 11.5 |
| *Level 3* | AP: Backward | 191 | 18.5 | 11.5 |
|  | ML: Right | 117 | 9.2 | 12.5 |
|  | AP: Forward | 191 | 18.5 | 11.5 |
|  | ML: Left | 117 | 9.2 | 13.5 |
| *Level 4* | ML: Right | 155 | 12.1 | 14.5 |
|  | AP: Backward | 255 | 23.9 | 12.5 |
|  | ML: Left | 155 | 12.1 | 14.5 |
|  | ML: Left (not rated) | 155 | 12.1 | 14.5 |
|  | AP: Forward | 255 | 23.9 | 14.5 |
| *Level 5* | AP: Forward | 319 | 29.5 | 14.5 |
|  | ML: Left | 192 | 15.1 | 17.5 |
|  | AP: Backward | 319 | 29.5 | 14.5 |
|  | ML: Right | 192 | 15.1 | 19.5 |
| *Level 6* | AP: Backward | 383 | 35 | 16.5 |
|  | ML: Right | 230 | 18 | 19.5 |
|  | AP: Forward | 383 | 35 | 16.5 |
|  | ML: Left | 230 | 18 | - |
| STT = Stepping Threshold Test, AP = anterior-posterior, ML = medio-lateral. | | | | |
