# Supplementary material for: The Stepping Threshold Test for assessing reactive balance discriminates between older adult fallers and non-fallers
Source: Front Sports Act Living. 2024 Oct 11;6:1462177. doi: 10.3389/fspor.2024.1462177 (PMC11502312; doi:10.3389/fspor.2024.1462177)
Supplement: Supplementary file 2 [file Table2.docx]

Supplementary Material

**Supplement 2: Scatter plots with regression line (solid line) and 95-% confidence intervals (dotted lines) for correlations of the all-step count evaluation strategy (STT-ACE; left column) and direction-sensitive evaluation strategy (STT-DSE; right column) of the Stepping Threshold Test (STT) with other assessments of fall risk factors.**

- Scatter plots A-H: Correlations of STT-ACE and STT-DSE with global (Brief Best Evaluation Systems Test, Brief-BESTest), static (balance test of the Short Physical Performance Battery, SPPB), dynamic (Four Square Step Test, FSST), and dynamic reactive balance (Dynamic Stepping Threshold Test, DSTT) (p. 2)
- Scatter plots I-P: Correlations of STT-ACE and STT-DSE with gait capacity (4-m gait speed test, GST; 4-meter gait speed test, 4-GST) and muscle strength (handgrip strength, HGS; 5-chair stand test, 5CST) (p. 3)
- Scatter plots Q-T: Correlations of STT-ACE and STT-DSE with functional capacity (SPPB, Timed Up and Go, TUG) (p. 4)
- Scatter plots U-Z: Correlations of STT-ACE and STT-DSE with fear of falling (Short Falls Efficacy Scale International, Short FES-I), global cognition (Mini-Mental State Examination, MMSE), and executive functioning (Trail Making Test B-A, TMT B-A) (p. 5)

| A  *r* = 0.56 (0.26; 0.77)  *p* < 0.001   | B  *r* = 0.69 (0.44; 0.84)  *p* < 0.001   |
| --- | --- |
| C  *r* = 0.53 (0.22; 0.74)  *p* = 0.001   | D  *r* = 0.64 (0.37; 0.81)  *p* < 0.001   |
| E  *r* = –0.37 (–0.64; –0.03)  *p* = 0.030   | F  *r* = –0.52 (–0.73; –0.20)  *p* = 0.002   |
| G  *r* = 0.58 (0.29; 0.77)  *p* < 0.001   | H  *r* = 0.68 (0.43; 0.84)  *p* < 0.001   |

| I  *r* = 0.38 (0.04; 0.64)  *p* = 0.028   | J  *r* = 0.44 (0.11; 0.68)  *p* = 0.009   |
| --- | --- |
| K  *r* = 0.50 (0.18; 0.72)  *p* = 0.003   | L  *r* = 0.62 (0.34; 0.79)  *p* < 0.001   |
| M  *r* = 0.18 (–0.17; 0.48)  *p* = 0.306   | N  *r* = 0.23 (–0.12; 0.54)  *p* = 0.185   |
| O  *r* = –0.22 (–0.53; 0.15)  *p* = 0.235   | P  *r* = –0.16 (–0.49; 0.21)  *p* = 0.378   |

| Q  *r* = 0.34 (0.00; 0.62)  *p* = 0.047   | R  *r* = 0.45 (0.12; 0.69)  *p* = 0.008   |
| --- | --- |
| S  *r* = –0.45 (–0.69; –0.12)  *p* = 0.008   | T  *r* = –0.55 (–0.76; –0.26)  *p* < 0.001   |

| U  *r* = –0.18 (–0.50; 0.18)  *p* = 0.300   | V  *r* = –0.28 (–0.57; 0.08)  *p* = 0.113   |
| --- | --- |
| W  *r* = 0.25 (–0.11; 0.55)  *p* = 0.158   | X  *r* = 0.46 (0.13; 0.69)  *p* = 0.007   |
| Y  *r* = –0.29 (–0.58; 0.06)  *p* = 0.091   | Z  *r* = –0.38 (–0.64; –0.04)  *p* = 0.027   |
