# Supplementary material for: The Stepping Threshold Test for assessing reactive balance discriminates between older adult fallers and non-fallers
Source: Front Sports Act Living. 2024 Oct 11;6:1462177. doi: 10.3389/fspor.2024.1462177 (PMC11502312; doi:10.3389/fspor.2024.1462177)
Supplement: Supplementary file 3 [file Table3.docx]

Supplementary Material

**Supplement 3: Sensitivity power analysis**

A sensitivity power analysis was conducted using G*Power 3.1 (Faul et al., 2007) to determine the minimum effect sizes that could be reliably detected by each statistical test used in this study (*t*-test for independent samples, Mann-Whitney *U* test, and *χ^2^*-test or Fisher’s exact test).

Given the sample size of *n*_1_ = 13 for fallers and *n*_2_ = 23 for non-fallers (STT: *n*_1_ = 12, *n*_2_ = 22), a statistical power (1−*β*) of 0.80, and a significance level (*α*) of 0.05, the sensitivity power analysis revealed that the study was adequately powered to detect large effect sizes with *t*-tests for independent samples (*d* = 1.00) and Mann-Whitney *U* tests (*d* = 1.03, STT: *d* = 1.06), and moderate to large effects with *χ^2^*-tests and Fisher’s exact tests (*w* = 0.46), which roughly correspond to an excellent AUC (≥0.8). Lower levels of discriminative accuracy may not have been as reliably detected, which should be considered when interpreting the AUC values.

Given the sample size of *n* = 34, a statistical power (1−*β*) of 0.80, and a significance level (*α*) of 0.05, the sensitivity power analyses revealed that the study was powered to detect moderate Spearman rank correlations (*r* = 0.46) for hypothesis testing related to the convergent validity of the STT. Lower correlations may not have been as reliably detected, so their interpretation also requires caution.

Reference:

Faul F, Erdfelder E, Lang AG, Buchner A. G*Power 3: a flexible statistical power analysis program for the social, behavioral, and biomedical sciences. Behav Res Methods. 2007;39(2):175-191.
